# MicroRNA-642b-3p functions as an oncomiR in gastric cancer by down-regulating the CUB and sushi multiple domains protein 1/smad axis

**DOI:** 10.1080/21655979.2022.2056813

**Published:** 2022-04-12

**Authors:** Haofeng Liu, Yuan Chen, Linsen Zhou, Xiaohui Jiang, Xiaojun Zhou

**Affiliations:** a Department of General Surgery, The First Affiliated Hospital of Soochow University, Suzhou P.R. China; b Department of General Surgery, Tumor Hospital Affiliated to Nantong University & Nantong Tumor Hospital, Nantong P.R. China; c Department of General Surgery, Yancheng First Hospital, Affiliated Hospital of Nanjing University Medical School, Yancheng P.R. China

**Keywords:** Gastric cancer, MicroRNA-642b-3p, CSMD1, smad signaling pathway, epithelial–mesenchymal transition, invasion, migration

## Abstract

Aberrant expression of microRNAs (miRNAs or miRs) has been involved in the progression of gastric cancer (GC). Our analysis of GC-related gene expression profiles identified the significantly up-regulated miR-642b-3p expression, which has been reported as a mediator in various cancers but rarely mentioned in researches on GC. Herein, this study intends to investigate the role of miR-642b-3p in GC development. Bioinformatics analysis was conducted to predict the downstream target gene of miR-642b-3p. Expression patterns of miR-642b-3p and CUB and sushi multiple domains protein 1 (CSMD1) in GC tissues and cell lines was then determined. Immunofluorescence, wound healing and Transwell invasion assays were performed to observe the malignant behaviors of GC cells with altered expression of miR-642b-3p and CSMD1. Nude mice with xenograft tumors were developed for *in vivo* validation. miR-642b-3p expression was increased in GC tissues and cell lines. miR-642b-3p targeted CSMD1 and reduced the expression of CSMD1, thereby inhibiting the activation of Smad signaling pathway. By this mechanism, the epithelial–mesenchymal transition (EMT), invasive and migratory potentials of GC cells were repressed. Meanwhile, *in vivo* data verified that miR-642b-3p enhanced the tumor growth of GC cells, which was associated with blockade of CSMD1-dependent activation of the Smad signaling pathway. Overall, miR-642b-3p acts as an oncomiR promoting tumor development in GC through suppressing CSMD1 expression and inactivating the Smad signaling pathway, which may enable the development of new therapeutic strategies for treatment of GC.

## Graphical abstract

**Figure:**
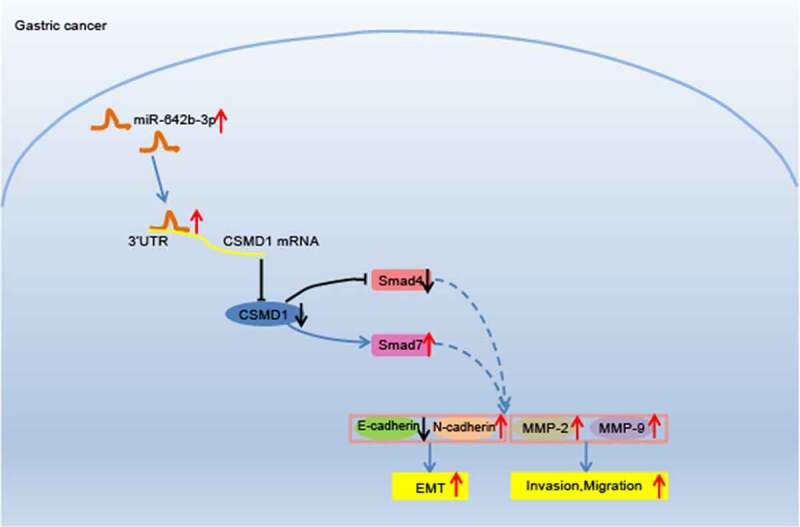

## Introduction

Gastric cancer (GC) represents the fourth most frequently occurring malignancy throughout the world, ranking second among the leading causes of mortality in relation to cancers [1]. Furthermore, GC is characterized by poor prognosis due to the significant metastatic potential and the high rate of recurrence, presenting a 5-year overall survival rate of less than 40% in most countries [2,3]. Thus, the delineation of molecular patterns behind GC progression is imperative for discovering novel antineoplastic protocols [4]. Notably, accumulating evidence has suggested that non-coding RNAs (ncRNAs) serve as modulator of gene expression implicated in the oncogenesis of GC [5–7], which sheds light on specific biomarkers for potential targeted therapy against the malignant phenotypes of GC [8].

As crucial ncRNAs, microRNAs (miRNAs or miRs) either suppress the translation of messenger RNAs (mRNAs) *via* binding to 3’-untranslated regions (3’-UTRs) or targeting protein-coding sequences [9]. More importantly, emerging data demonstrated that miRNAs function as cancer ‘drivers’ due to the vital role of their alteration in modulating malignant transformation and progression [10], including GC [11,12]. During our previous analysis of GC-related gene expression profiles, miR-642b-3p was noticed for its significantly up-regulated expression. Interestingly, miR-642b-3p was rarely investigated in GC studies even though it has been highlighted as mediator of genes at transcriptional levels in colorectal cancer [13], and pancreatic cancer [14]. Accordingly, exploring the specific role of miR-642b-3p in GC may lay a theoretical foundation for the development of novel targeted therapies for GC.

Further, CUB and sushi multiple domains protein 1 (CSMD1) was suggested to be the putative target gene of miR-642b-3p through our bioinformatics analysis. Accumulating evidence supports that reduced expression of CSMD1 has a bearing on unsatisfactory prognosis in a variety of cancers [15–17]. A previous study has also manifested that the deregulation of CSMD1, targeted by specific miRNAs, triggers the progression of GC [18]. In addition, the anti-tumor activity of CSMD1 has been associated with activation of the Smad signaling pathway [19]. Taken together, we proposed a hypothesis that miR-642b-5p might directly mediate the CSMD1 gene and modulate the Smad signaling pathway, leading to its regulatory function in GC.

## Materials and methods

### Ethics statement

This study was approved by the Ethics Committee of the Tumor Hospital Affiliated to Nantong University & Nantong Tumor Hospital and the written informed consents were provided by all participants or legal guardians. Animal experiments were approved by the Animal Care and Use Committee of the Tumor Hospital Affiliated to Nantong University & Nantong Tumor Hospital (Approval number: 2017–075).

### Bioinformatics analysis

The expression profiles of miRNA related to GC were retrieved from the Gene Expression Omnibus (GEO) database (https://www.ncbi.nlm.nih.gov/gds/). Differential expression analysis was performed utilizing Limma package of R language software, followed by plotting a heat map of differential expression of miRNAs through a Pheatmap package. MiR-642b-3p target genes were predicted using the TargetScan (http://www.targetscan.org/vert_72/) and the miRDB (http://mirdb.org) databases. A Venn diagram of predicted target genes was subsequently constructed [20].

### Tissue collection

Samples were collected from 30 patients (19 males and 11 females; aged 59.47 ± 8.18 years) with GC initially diagnosed at the Department of Stomach Oncology of the Tumor Hospital Affiliated to Nantong University & Nantong Tumor Hospital from March 2018 to November 2020. The samples consisted of 13 cases of stage I GC, 14 cases of stage II, and 3 cases of stage III. None of these participants received radiotherapy or chemotherapy before surgery. The histopathological types of the samples were determined by reference to the WHO pathological classification [21]. Tumor tissues along with adjacent normal gastric mucosa tissues (≥5 cm from the edge of tumors), confirmed by histopathological examination, were harvested. The extracted tissues were stored in liquid nitrogen for subsequent analyses.

### Cell line screening

Five human GC cell lines, SNU-5, AGS, MKN-74, HGC-27, and KATO III and the normal gastric mucosal epithelial cell line GES-1 were purchased from Wuhan Procell Life Science & Technology (Wuhan, China). These cells were recovered and cultured in Roswell Park Memorial Institute (RPMI)-1640 medium (Gibco, Grand Island, NY) supplemented with 10% FBS, penicillin (100 U/mL), and streptomycin (100 mg/mL) under saturated humidity, 5% CO_2_, and 37°C. When cell confluence reached 90%, serial passaging was performed. The cells were detached with 0.25% trypsin until the cells become round and gaps appeared, which was subsequently terminated by addition of FBS culture solution. Next, the cells were pipetted into a single-cell suspension. Quantitative real-time polymerase-chain reaction (qRT-PCR) was then employed to determine miR-642b-3p expression in GC cell lines [22].

### qRT-PCR

Total RNA was extracted by TRIzol reagent (15,596,026, Invitrogen). RNA integrity was characterized utilizing agarose gel electrophoresis (1%), and RNA concentration and purity were measured utilizing ND-1000 spectrophotometer. Based on protocols of PrimeScript RT reagent Kit (RR047A, Takara, Dalian, China), RNA was reverse-transcribed into cDNA in a 20 μL reaction system. For miR-642b-3p and U6 detection, RNA was reverse-transcribed into cDNA using microRNA Reverse Transcription Kit (EZ Bioscience, EZB-miRT2-S). The primers for miR-642b-3p, Smad7, Smad4, CSMD1, alpha smooth muscle actin (α-SMA), fibronectin (FN), matrix metallopeptidase (MMP)-2, MMP-9, U6, and GAPDH were synthesized commercially (Sangon, Shanghai, China), as shown in Supplementary Table 1. qRT-PCR was performed following protocols of SYBR® Premix Ex Taq^TM^ II kit (TaKaRa) and ABI 7500 PCR system (Applied Biosystems, Foster City, CA). The relative expression of target genes was calculated based on the 2^−ΔΔCt^ method [23], with U6 and GAPDH serving as housekeeper genes.

### Western blot

Total protein was isolated from the collected cells by RIPA lysis buffer (P0013B, Beyotime, Shanghai, China). Protein concentration, measured utilizing the BCA protein quantification kit (Beyotime), was adjusted to 4 µg/µL with phosphate-buffered saline (PBS). Then, 30 μg total protein was dissolved by sodium dodecyl sulfate-polyacrylamide gel electrophoresis (SDS-PAGE). Separated proteins were transferred to nitrocellulose membrane. Blots were probed with diluted primary antibodies (Abcam, Cambridge, UK), including Smad7 (ab190987, 1:2000), Smad4 (ab40759, 1:5000), CSMD1 (ab166908, 1:1000), α-SMA (ab5694, 1:1000), FN (ab2413, 1:1000), MMP-2 (ab92536, 1:1000), MMP-9 (ab73734, 1:1000), GAPDH (ab9485, 1:2500, internal reference) at 4°C overnight on a shaker, followed by 2-h incubation with diluted horseradish peroxidase (HRP)-labeled secondary antibody goat anti-rabbit immunoglobulin G (IgG; ab205718, 1:2000–1:50,000) at room temperature. Blots were visualized by enhanced chemiluminescence (ECL) reagent [24]. Images were photographed and analyzed using the Quantity One software.

### Dual-luciferase activity assay

Wild type (WT) sequence of CSMD1 mRNA 3’-UTR and the mutant (Mut) sequence were synthesized. The pmiR-RB-REPORT^TM^ plasmid (RiboBio, Guangzhou, Guangdong, China) was digested by restriction endonucleases, and then WT and Mut were constructed, respectively, into pmiR-RB-REPORT^TM^ (Guangzhou RiboBio) along with miR-642b-3p mimic and mimic negative control (NC). The correctly sequenced luciferase reporter plasmids carrying WT and Mut were selected for subsequent transfection. Transfected cells were lysed and centrifuged for 3–5 min. The supernatant was collected and the luciferase activity was measured using Luciferase Assay Kit (RG005, Beyotime). The relative luciferase activity was calculated as the ratio of relative luciferase activity of firefly luciferase to that of Renilla luciferase [25].

### Cell culture and transfection

SNU-5 cell line was recovered and cultured in RPMI-1640 medium containing 10% calf serum (renewed every 3 days) in an incubator (5% CO_2_, 37°C). Cells were classified and, respectively, subjected to treatments of miR-642b-3p mimic, miR-642b-3p inhibitor, CSMD1 overexpression plasmid, Smad signaling pathway inhibitor SB431542 (Abcam, ab120163, treatment concentration of 10 μM). The aforementioned plasmids were purchased from Dharmacon Company (Lafayette, CO).

### RNA delivery by lentivirus vector

The 293 T cells (American Type Culture Collection [ATCC; Manassas, VA]; cultured in DMEM medium containing 10% FBS and 1% penicillin-streptomycin) were seeded in six-well plates (10^5^ cells/well). Cells grown to 80% confluence were transfected with polyethylenimine (PEI, Solarbio, Beijing, China). Then, 10 μg of the lentiviral vector protocadherin (pCDH) with desired plasmids, 7.5 μg of auxiliary plasmid paired box containing (PAX), and 5 μg of pMD2.G were diluted in a 750 μL reduced-serum opti-minimum essential medium (MEM, Gibco), which was allowed to stand for 5 min after mixed well. Further, 112.5 μg of PEI was diluted and mixed, followed by standing at ambient temperature for 5 min. The mixture was incubated with DMEM (37°C, 5% CO_2_). The medium was replaced by complete medium (8 mL) 6 h later for another 48-h culture, and cell supernatant was then collected, followed by 5-min centrifugation (800 g) and filtration with a 0.45 μm filter. After that, 30 mL filtrate was mixed with 7.5 mL 5× PEG8000 mother liquor every 30 min, with 3–5 replications, and leaved to stand at 4°C overnight. The supernatant was removed after 40-min centrifugation (4000 g, 4°C), and the precipitate was resuspended in 1 mL DMEM.

The 293 T cells in good conditions were digested, centrifuged a day in advance, and resuspended in complete medium. The suspension was seeded in 24-well plates (8,000 cells/well). On the second day, the medium in the 24-well plate was aspirated, and 500 μL of complete medium (containing 0.05, 0.5, 5, and 50 μL lentivirus) was successively added to each well before 72-h incubation in a CO_2_ incubator. The 293 T cells in each well were digested. The proportion of green fluorescent protein (GFP)-positive cells was measured by flow cytometry, and the titer of virus was calculated according to the formula: Virus titer (TU/mL) = (A + B × 10) × 1000/2/A, A (the number of visible fluorescence in the penultimate), B (the number of fluorescence visible in the last).

The human GC cell line SNU-5 was seeded six-well plates (3 × 10^5^ cells/well) and cultured. When reaching 50–80% confluence, cells were transduced with lentivirus for the preparation of stable cell lines using Polybrene kit (Sigma, St. Louis, MS) [26].

### Immunofluorescence staining

The cells of 70% confluence were incubated with E-cadherin antibody (rabbit anti-human, Abcam, ab40772, 1:500) and N-cadherin antibody (rabbit anti-human, Abcam, ab18203, 1:5000) in a refrigerator (4°C) overnight. Further, cells were subjected to 2-h incubation with fluorescence-labeled secondary antibody IgG (goat anti-rabbit, ab205718, 1:2000–1:50,000) without light exposure at room temperature. Then, cells were stained with DAPI (1:10, Vector Laboratories, Burlingam, CA) for microscopic examination [27].

### Wound healing assay

Horizontal lines were drawn uniformly by a marker on the back of 6-well plates. Cells were trypsinized and pipetted into single-cell suspension for cell counting. Cells were seeded into 6-well plates (1 × 10^6^ cells/well), followed by 24 h of incubation in complete medium. Subsequently, the medium was replaced by 10% FBS-containing RPMI-1640 medium. Lines perpendicular to the horizontal lines were scratched by a sterilized 10-µL micropipette tip. The plate was added with serum-free medium for incubation (37°C, 5% CO_2_). Images were photographed during observation utilizing a microscope (Olympus, Tokyo, Japan) at 0 and 24 h [28].

### Transwell invasion assay

Matrigel (Sigma) was thawed overnight at 4°C and diluted with serum-free RPMI-1640 medium to a final concentration of 1 mg/mL. The diluted Matrigel was vertically added into the bottom center of the Transwell upper insert (8 µm pore size) by 80 µL/well. Next, 700 µL of RPMI-1640 medium was added to the lower insert, and cell suspension (1 × 10^6^ cells/mL) was added to the upper. The plate was cultured in the incubator for 24 h. The insert was fixed with 4% paraformaldehyde (30 min) and stained with 0.05% crystal violet (room temperature, 30 min). Cells in the upper insert were wiped off with a wet cotton swab. Transwell plates were then observed with an inverted microscope [29].

### Enzyme-linked immunosorbent assay (ELISA)

Cells were digested with 0.25% trypsin, dispersed into a single-cell suspension, counted, and seeded in 6-well plates (1 × 10^6^ cells/well). After culture in complete medium for 24 h. the cell culture supernatant was harvested in which MMP-2 and MMP-9 protein levels were determined using human MMP-2 ELISA kit (ab267813, Abcam) and human MMP-9 ELISA kit (ab246539, Abcam) [30].

### Tumor xenografts in nude mice

A total of 30 five-week-old male BALB/c nude mice (weighing 18–22 g; Beijing Vital River Laboratory Animal Technology, Beijing, China) were housed individually in the specific pathogen-free (SPF) laboratory at 22–25°C and 60–65% humidity under a 12-h light/dark cycle. The mice were acclimated for one week before experiment. The health of the mice was observed before the experiment. The mice were randomly divided into 5 groups (6 mice/group), subcutaneously injected with SNU-5 cells (1 × 10^6^) transfected with miR-642b-3p mimic NC, miR-642b-3p mimic, CSMD1, CSMD1 NC or CSMD1 + miR-642b-3p mimic. Following successful inoculation, observations of the xenograft tumor formation were made on the 7th, 14th, 21st, 28th, and 35th days, by recording the long diameter (a) and short diameter (b) of the tumor. The volumes of tumors were then calculated based on the formula: volume = (a × b^2^)/2. The nude mice were euthanized on the 35th day by anesthesia with overdose of sodium pentobarbital. The intact xenograft tumor of each mouse was removed and weighed [31].

### Immunohistochemical detection of microvessel density

Streptavidin-peroxidase (SP) conjugate method was adopted for immunohistochemical staining. Prepared 5-μm slides of tumor tissues were dewaxed, hydrated, and heated for 10 min to retrieve antigen. The endogenous peroxidase activity was quenched by 3% H_2_O_2_. The slides were blocked with 5% bovine serum albumin (BSA)/TBST. After that, the slides were incubated with rabbit anti-human primary antibodies against CD34 (ab81289, 1:100, Abcam) at room temperature for 4 h and then with secondary antibody donkey anti-rabbit IgG (ab150073, 1:1000, Abcam) at ambient temperature for 15 min. HRP-labeled streptavidin was added the same way. DAB was added to develop color. The slides were further stained with hematoxylin and observed under a microscope (BX43, Olympus). The microvessel density was determined using the Weidner counting method [32].

### Statistical analysis

Data were processed utilizing SPSS 22.0 software (IBM Corp., Armonk, NY). Measurement data were presented as mean ± standard deviation. Data between GC tissues and adjacent normal tissues were compared by paired t test. The comparison between two variables was performed by independent sample *t*-test [33]. Data among multiple groups were compared by one-way analysis of variance (ANOVA) with Tukey’s post hoc test [33]. Tumor volume at various time points was compared by repeated measures ANOVA with Tukey’s post hoc test. *p* < 0.05 refers to statistically significant difference.

## Results

In this study, we validated that miR-642b-3p is abundantly expressed in GC tissues and cells. We also found that miR-642b-3p targets CSMD1 to induce EMT, migration and invasion of GC cells. Furthermore, CSMD1 activates Smad to inhibit EMT, migration and invasion of GC cells, while miR-642b-3p promotes GC growth by regulating the CSMD1/Smad signaling. Our study provides therapeutic targets for treatment of GC.

### Bioinformatics analysis reveals that miR-642b-5p and its downstream target gene CSMD1 have regulatory effects on GC

**Figure 1.**
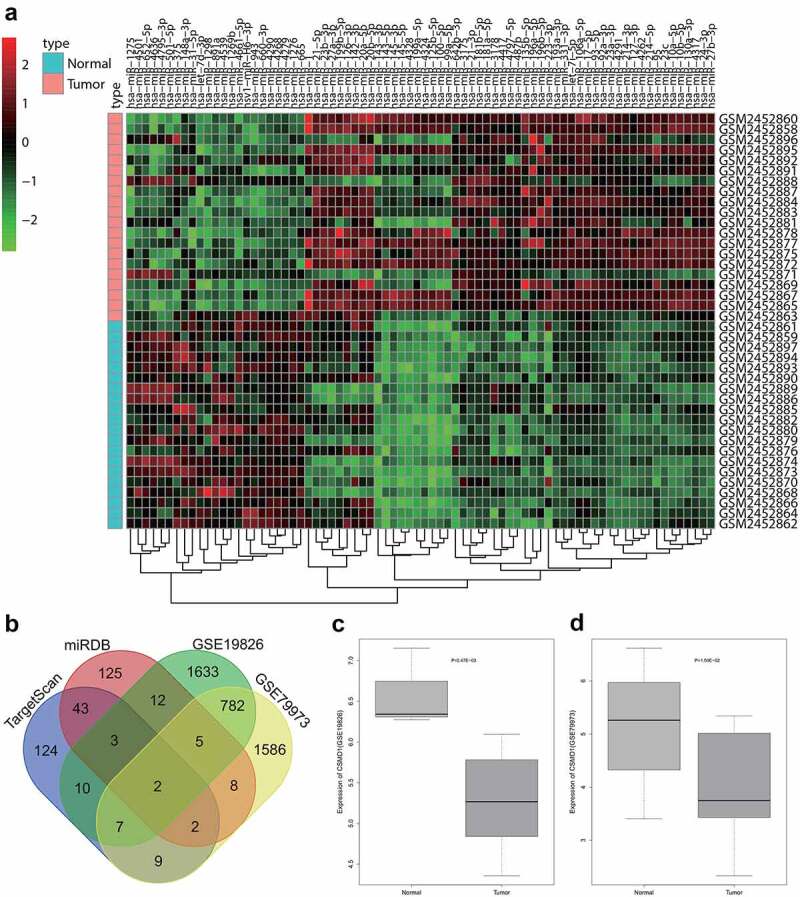
Bioinformatics analysis predicts GC-related differentially expressed miRNAs and their potential downstream mRNAs. A: A heat map of differential expression of miRNAs in GC samples based on GC-related miRNA expression dataset GSE93415. B: The intersection of predicted target genes of miR-642b-3p by the TargetScan and miRDB databases and down-regulated genes in GC samples in the GC-related gene expression datasets GSE19826 and GSE79973. C, The expression of CSMD1 in GC samples in the GSE19826 dataset. D: The expression of CSMD1 in GC samples in the GSE79973 dataset.

Differential analysis was first performed on the GSE93415 miRNA dataset related to GC retrieved from GEO database suggested 76 differentially expressed miRNAs in GC samples, including 53 highly expressed and 23 poorly expressed miRNAs (Figure 1a). It is also noted that miR-642b-3p exhibited significant high expression in GC cells. Previous studies have identified the critical regulatory function of miR-642b-3p in various cancers [14,34,35], while the effect of miR-642b-3p was seldom reported in the pathogenesis of GC.

To unveil the possible role of miR-642b-3p in GC, we utilized the TargetScan and miRDB databases to predict the potential downstream target genes of miR-642B-3p. After the differential expression analysis of two GC-related microarrays (GSE19826 and GSE79973), the genes with significantly reduced expression in GC were overlapped with the predicted target genes of miR-642b-3p (Figure 1b). Two genes, namely CSMD1 and epididymal protein 3A (EDDM3A), fell into the intersection. The expression of CSMD1 was down-regulated in GSE19826 and GSE79973 expression profiles (Figure 1c, d).

Furthermore, we searched on the function of the two genes CSMD1 and EDDM3A and found almost no report about the role of EDDM3A gene in tumor regulation. In contrast, increasing studies have indicated that the CSMD1 gene is involved in the progression of colorectal cancer [36], breast cancer [16] and other tumors [37]. It has also been suggested that CSMD1 can impede tumor formation through the Smad signaling pathway, which has a role to confer in the pathophysiological processes of GC [19,38,39].

Taken together, miR-642b-3p is likely to directly mediate the CSMD1 gene and thereby regulate the Smad signaling pathway to exert its regulatory function in GC.

### miR-642b-3p expression is up-regulated in GC tissues and cells

**Figure 2.**
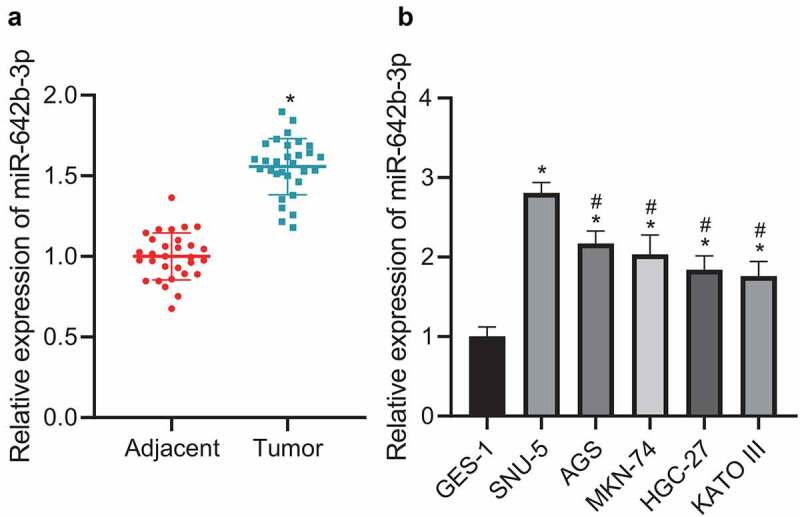
miR-642b-3p is highly expressed in GC tissues and cells. A: miR-642b-3p expression in GC tissues (n = 30) and adjacent normal tissues (n = 30) determined by qRT-PCR; * *p* < 0.05 versus the adjacent normal tissues; B: miR-642b-3p expression in normal gastric mucosal epithelial cell line GES-1 and GC cell lines SNU-5, AGS, MKN-74, HGC-27 and KATO III determined by qRT-PCR; * *p* < 0.05 versus the GES-1 cell line, # *p* < 0.05 versus the SNU-5 cell line. Measurement data were presented as mean ± standard deviation. Data between two groups were performed by independent sample *t*-test. Data among multiple groups were compared by one-way ANOVA with Tukey’s post hoc test. The cell experiment was repeated three times independently.

The aforementioned results suggested a role of miR-642b-3p in the pathogenesis of GC. Further, we moved to validating miR-642b-3p expression in GC tissues and cells. qRT-PCR results (Figure 2a) revealed that miR-642b-3p was highly expressed in GC tissues, versus adjacent normal tissues. Relative to GES-1 cells, miR-642b-3p expression in GC cell lines (SNU-5, AGS, MKN-74, HGC-27 and KATO III) was elevated, among which SNU-5 cell line presented with the highest expression of miR-642b-3p (Figure 2b) and was thus selected for subsequent experiments. These data indicated the increased miR-642b-3p expression in GC tissues and cells.

### miR-642b-3p contributes to down-regulation of CSMD1 and stimulates the malignant phenotypes of GC cells

**Figure 3.**
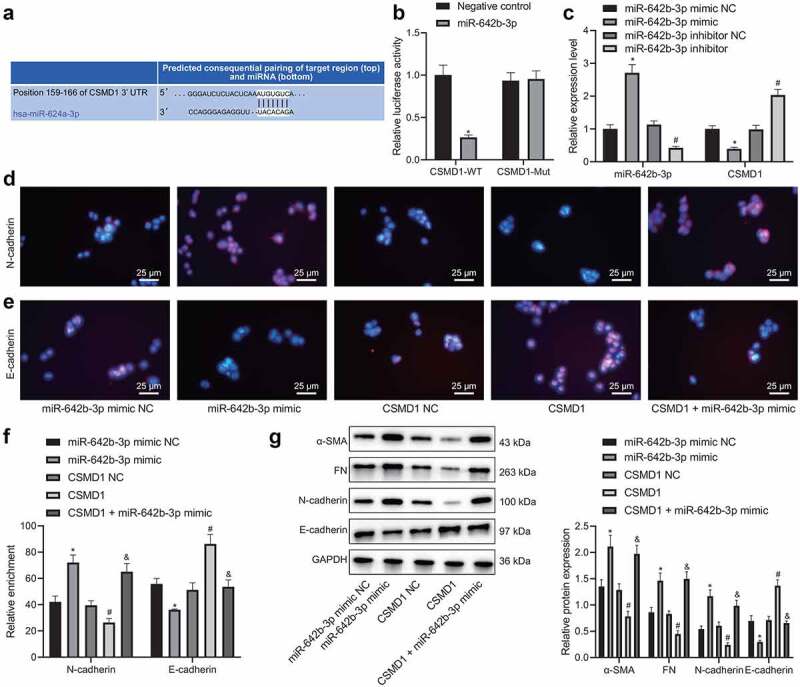
miR-642b-3p down-regulates the expression of CSMD1 and suppresses the EMT of GC cells. A: The predicted binding sites between miR-642b-3p and CSMD1; B: The binding between miR-642b-3p and CSMD1 verified by dual luciferase reporter assay. C: miR-642b-3p and CSMD1 expression in SNU-5 cells transfected with miR-642b-3p mimic or miR-642b-3p inhibitor determined by qRT-PCR; D: Immunofluorescence staining images of N-cadherin protein in SNU-5 cells treated with miR-642b-3p mimic, CSMD1 or both; E: Immunofluorescence staining images of E-cadherin in SNU-5 cells treated with miR-642b-3p mimic, CSMD1 or both; F: Quantitative analysis of the positive rate of N-cadherin and E-cadherin proteins in SNU-5 cells treated with miR-642b-3p mimic, CSMD1 or both; G: α-SMA, FN, N-cadherin and E-cadherin protein expression in SNU-5 cells treated with miR-642b-3p mimic, CSMD1 or both determined by western blot normalized to GAPDH. * *p* < 0.05 versus the miR-642b-3p mimic NC group, # *p* < 0.05 versus the CSMD1 NC group, & *p* < 0.05 versus the CSMD1 group. Measurement data were presented as mean ± standard deviation. Data among multiple groups were compared by one-way ANOVA with Tukey’s post hoc test. The cell experiment was repeated three times independently.

We then proceeded to validate the interaction between miR-642b-3p and CSMD1. Online prediction software predicted the presence of binding sites between miR-642b-3p and CSMD1 (Figure 3a). Meanwhile, dual-luciferase reporter assay results showed that the luciferase activity of CSMD1-WT was reduced upon transfection with miR-642b-3p mimic whereas that of CSMD1-MUT exhibited no obvious changes (Figure 3b), indicating the potential targeting between miR-642b-3p and CSMD1. In addition, qRT-PCR results revealed a decline in the expression of CSMD1 yet an increase in that of miR-642b-3p in SNU-5 cells transfected with miR-642b-3p mimic. Conversely, opposite results were noted in the presence of miR-642b-3p inhibitor (Figure 3c). These results indicate that miR-642b-3p may target CSMD1 and downregulate its expression.

According to the results of immunofluorescence assay (Figure 3d-f), the positive rate of E-cadherin protein was reduced while that of N-cadherin was increased in the SNU-5 cells transfected with miR-642b-3p mimic. However, the positive rate of E-cadherin protein was increased but that of N-cadherin protein was reduced in the presence of CSMD1 overexpression. Moreover, the effect of CSMD1 overexpression alone was reversed by further miR-642b-3p mimic.

Besides, miR-642b-3p mimic elevated the protein expression of epithelial–mesenchymal transition (EMT)-related α-SMA, N-cadherin and FN but decreased that of E-cadherin in SNU-5 cells (Figure 3g). Opposite results were found in SNU-5 cells overexpressing CSMD1. Moreover, the simultaneous overexpression of CSMD1 and miR-642b-3p led to higher protein expression of α-SMA, N-cadherin and FN yet lower protein expression of E-cadherin compared with individual CSMD1 overexpression. Based on the above results, a conclusion is drawn that miR-642b-3p can reduce CSMD1 expression and thus induce EMT of GC cells.

**Figure 4.**
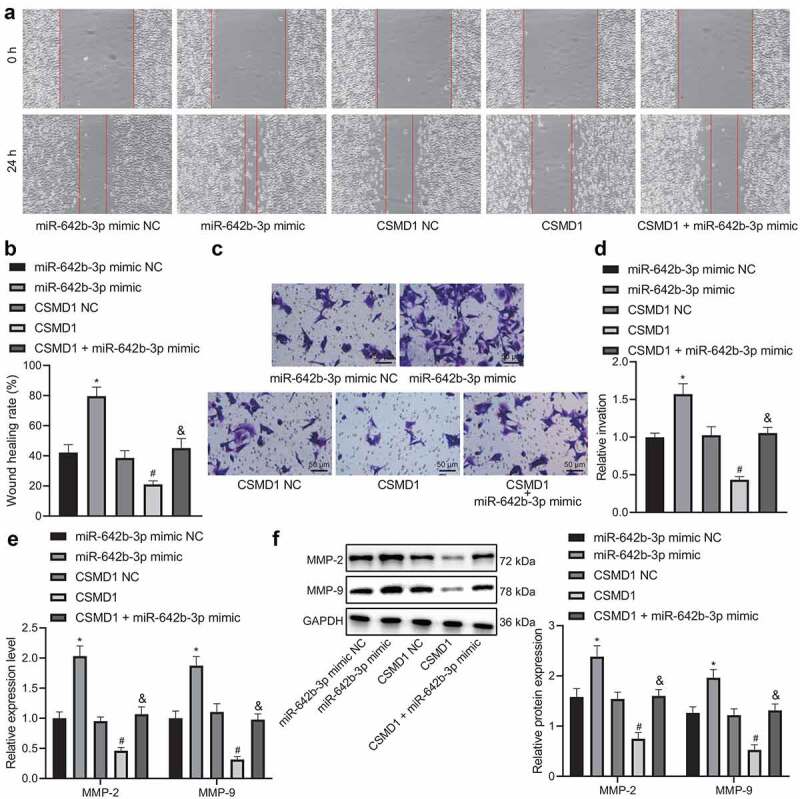
miR-642b-3p down-regulates the expression of CSMD1 and impedes the migration and invasion of GC cells. A: Representative images of migration of SNU-5 cells treated with miR-642b-3p mimic, CSMD1 or both measured by wound healing assay; B: Quantitative analysis of panel A; C: Representative images of invasion of SNU-5 cells treated with miR-642b-3p mimic, CSMD1 or both measured by Transwell assay; D: Quantitative analysis of panel C; E: qRT-PCR detection of relative mRNA expression of metastasis-related genes MMP-2 and MMP-9 in SNU-5 cells treated with miR-642b-3p mimic, CSMD1 or both; F: Protein expression of metastasis-related genes MMP-2 and MMP-9 in SNU-5 cells treated with miR-642b-3p mimic, CSMD1 or both as determined by western blot normalized to GAPDH. Measurement data were presented as mean ± standard deviation. Data among multiple groups were compared by one-way ANOVA with Tukey’s post hoc test. * *p* < 0.05 versus the miR-642b-3p mimic NC group, # *p* < 0.05 versus the CSMD1 NC group, & *p* < 0.05 versus the CSMD1 group. The cell experiment was repeated three times independently.

Furthermore, the results of wound healing assay (Figure 4a, b), Transwell assay (Figure 4c, d), qRT-PCR (Figure 4e) and Western blot (figure 4f) demonstrated that miR-642b-3p mimic augmented the migration and invasion of SNU-5 cells, corresponding to increased expression of metastasis-related genes MMP-2 and MMP-9. However, opposite results were found in SNU-5 cells overexpressing CSMD1. In addition, CSMD1 in combination with miR-642b-3p mimic led to an elevation in the migration and invasion of SNU-5 cells, along with the higher expression of MMP-2 and MMP-9 genes, as compared to CSMD1 overexpression alone. Meanwhile, ELISA data revealed the consistent results in the expression of MMP-2 and MMP-9 proteins in the SNU-5 cell supernatant as those of western blot (Supplementary Figure 1A).

Therefore, these data suggest that miR-642b-3p downregulates CSMD1 expression and attenuates migratory and invasive abilities of GC cells.

### Overexpression of CSMD1 activates the Smad signaling pathway and arrests EMT, migration and invasion of GC cells

**Figure 5.**
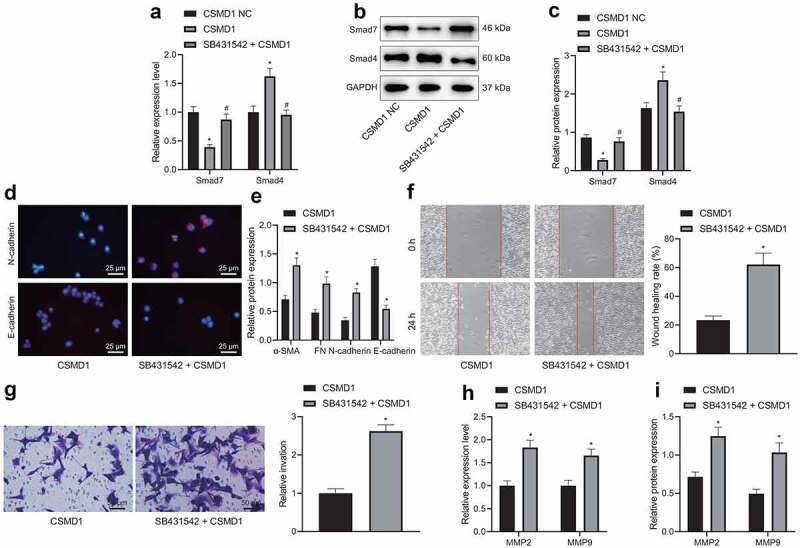
Overexpression of CSMD1 promotes activation of Smad signaling pathway, thus suppressing the EMT, migration and invasion of GC cells. A: qRT-PCR detection of Smad7 and Smad4 mRNA expression in SNU-5 cells treated with CSMD1 or combined with SB431542; B: Representative western blots of Smad7 and Smad4 proteins in SNU-5 cells treated with CSMD1 or combined with SB431542; C: Quantification of Smad7 and Smad4 protein expression in SNU-5 cells treated with CSMD1 or combined with SB431542; In panel A-C, * *p* < 0.05 versus the CSMD1 group, # *p* < 0.05 versus the CSMD1 group. D: Immunofluorescence staining images of N-cadherin and E-cadherin proteins in SNU-5 cells treated with CSMD1 or combined with SB431542; E: α-SMA, FN, N-cadherin and E-cadherin protein expression in SNU-5 cells treated with CSMD1 or combined with SB431542 determined by western blot; F: Migration of SNU-5 cells treated with CSMD1 or combined with SB431542 measured by wound healing assay; G: Invasion of SNU-5 cells treated with CSMD1 or combined with SB431542 measured by Transwell assay; H: qRT-PCR detection of relative mRNA expression of metastasis-related genes MMP-2 and MMP-9 in SNU-5 cells treated with CSMD1 or combined with SB431542; I: Protein expression of metastasis-related genes MMP-2 and MMP-9 in SNU-5 cells treated with CSMD1 or combined with SB431542 as determined by western blot. * *p* < 0.05 versus the CSMD1 NC group. Measurement data were presented as mean ± standard deviation. Data among multiple groups were compared by one-way ANOVA with Tukey’s post hoc test. The cell experiment was repeated three times independently.

The next focus of this study was to examine the downstream mechanism of CSMD1 affecting the biological functions of GC cells. Overexpression of CSMD1 in SNU-5 cells led to a decrease in Smad7 expression and an increase in Smad4 expression; and further treatment with SB431542 abrogated the effect of CSMD1 on the expression of Smad7 and Smad4 (Figure 5a, b,c). Together, overexpression of CSMD1 can activate the Smad signaling pathway.

Immunofluorescence assay indicated lower positive rate of E-cadherin protein and higher positive rate of N-cadherin protein in the SNU-5 cells treated with SB431542 + CSMD1 than CSMD1 alone (Figure 5d). Western blot data exhibited that SB431542 reversed the promoting effect of CSMD1 on E-cadherin expression and the inhibiting effect on that of α-SMA, N-cadherin and FN (Figure 5e).

In addition, treatment with SB431542 + CSMD1 promoted the migration (figure 5f) and invasion (Figure 5g) of SNU-5 cells relative to individual treatment with CSMD1, as further shown by up-regulated expression of MMP-2 and MMP-9 (Figure 5h, i). As depicted in Supplementary Figure 1B, ELISA data revealed consistent results in the expression of MMP-2 and MMP-9 proteins in the SNU-5 cell supernatant with those of western blot.

Altogether, these data demonstrate that the inhibiting property of CSMD1 on the EMT, migration and invasion of GC cells may be closely related to activation of the Smad signaling pathway.

### miR-642b-3p downregulates CSMD1 and inactivates the Smad signaling pathway, thus promoting the tumor growth of GC cells in vivo

**Figure 6.**
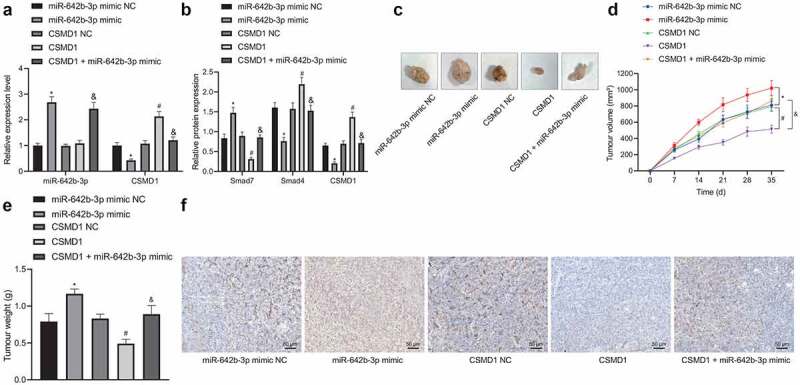
miR-642b-3p downregulates CSMD1 to inactivate the Smad signaling pathway, thereby enhancing the tumor growth of GC cells in nude mice. A: qRT-PCR detection of miR-642b-3p and CSMD1 expression in the tumor tissues of mice treated with miR-642b-3p mimic, CSMD1 or both; B: CSMD1, Smad4 and Smad7 protein expression in the tumor tissues of mice treated with miR-642b-3p mimic, CSMD1 or both; C: Representative photographs of the xenograft tumors in the nude mice treated with miR-642b-3p mimic, CSMD1 or both; D: Quantitative analysis of volume of the xenograft tumor in the nude mice treated with miR-642b-3p mimic, CSMD1 or both; E: Quantitative analysis of weight of the xenograft tumor in the nude mice treated with miR-642b-3p mimic, CSMD1 or both; F: Microvessel density of tumors in nude mice treated with miR-642b-3p mimic, CSMD1 or both as detected by immunohistochemical assay. n = 6. * *p* < 0.05 versus the miR-642b-3p mimic NC group, # *p* < 0.05 versus the CSMD1 NC group, & *p* < 0.05 versus the CSMD1 group. Measurement data were presented as mean ± standard deviation. Data among multiple groups were compared by one-way ANOVA with Tukey’s post hoc test. Data at various time points were compared by repeated measures ANOVA with Tukey’s post hoc test.

Finally, we aimed to characterize the effect of miR-642b-3p regulating the CSMD1/Smad signaling on the growth of GC in vivo. qRT-PCR and Western blot results (Figure 6a-b) exhibited abundant expression of miR-642b-3p and Smad7 yet poor expression of CSMD1 and Smad4 in the tumor tissue of mice treated with miR-642b-3p mimic, the effects of which were undermined by CSMD1. Higher expression of miR-642b-3p and Smad7 yet lower expression of CSMD1 and Smad4 were noted in the presence of CSMD1 + miR-642b-3p mimic than CSMD1 alone.

Additionally, tumor volume and weight were increased in mice treated with miR-642b-3p mimic but they were decreased upon treatment with CSMD1. Versus CSMD1 alone, CSMD1 + miR-642b-3p mimic resulted in more obvious increase in tumor volume and weight (Figure 6c-e). Meanwhile, immunohistochemical staining results showed that the microvessel density of subcutaneously transplanted tumors in the miR-642b-3p mimic-treated mice was elevated. However, CSMD1 treatment decreased the microvessel density, which was negated in response to treatment with CSMD1 + miR-642b-3p mimic (figure 6f).

The above results indicate that miR-642b-3p inactivates the Smad signaling pathway by downregulating the expression of CSMD1, thereby accelerating the tumor growth of GC cells in vivo.

## Discussion

Accumulating evidence has suggested that miRNAs have a vital role to play in modulating the malignant transformation of GC [12,40]. Further, our preliminary analysis of GC-related gene expression profiles identified the obviously up-regulated expression of miR-642b-3p, which has been highlighted as a mediator of genes at transcriptional levels in a variety of cancers [13,14]. Subsequently, CSMD1, a frequently reported tumor suppressor [15,16,41], was suggested to be the putative target gene of miR-642b-3p by bioinformatics analysis. Therefore, in this investigation, we illustrated that miR-642b-3p directly bound to CSMD1 gene, thereby exerting tumor-promoting function in GC.

Our initial finding of the significant overexpression of miR-642b-3p in GC tissues and cells indicated that miR-642b-3p might participate in the carcinogenesis of GC. This finding corroborates an existing study on the abnormal high expression of miR-642b-3p in pancreatic cancer, where plasma level of miR-642b-3p has been proposed to serve as a diagnostic target in low-grade tumor stage [14]. There is a paucity of data underlining the importance of regulatory function of miRNAs in GC, which delineated that the up-regulated expression of several miRNAs, such as miR-193a3p [42], miR-221, and miR-222 [43], were involved in the malignant phenotypes of GC cells. The aberrant overexpression of miR-642b-3p has also been documented in carcinogenesis of non-small cell lung cancer, which influences cancer cell behaviors through interplay with target genes [34]. Moreover, since the discovery of miRNAs, their role as endogenous repressor of numerous target genes has been identified [44,45]. In this sense, we then moved to the observation of the downstream genes of miR-642b-3p, to further unveil the underlying molecular mechanism. Our bioinformatics analysis showed that miR-642b-3p targeted and inversely modulated CSMD1. In other words, the mediatory effect of miR-642b-3p might be achieved through directly binding to CSMD1 gene at transcriptional level. Such hypothesis conforms to a previous study that the deregulation of CSMD1 targeted by miR-10b could trigger the progression of GC [18]. Additionally, the tumor-inhibiting role of CSMD1 has been established in various cancers, including breast cancer [46], colorectal cancer [17], and hepatocellular carcinoma [47]. Based on the above experimental results and evidences, we drew a conclusion that miR-642b-3p targeting CSMD1 gene served as the precondition for the pro-tumorigenic activities of miR-642b-3p in GC progression. Further, we validated the effect exerted by miR-642b-3p and CSMD1 on GC through artificial modulation of their expression.

It was demonstrated that silencing miR-642b-3p or up-regulating CSMD1 could suppress the EMT of GC cells and further impede their migratory and invasive potential, as reflected by elevated E-cadherin expression and repressed N-cadherin, MMP-2 and MMP-9 expression. Meanwhile, the activation of Smad signaling pathway was found to be augmented by diminishing miR-642b-3p or restoring CSMD1. Of note, EMT is a complex process presenting a pivotal role in the invasion and metastasis of tumors involving reduced cellular adhesion and facilitated cellular mobility [48]. Taken together, our experiment established that the deregulation of CSMD1 targeted by miR-642b-3p might drive GC progression through the Smad signaling pathway. These findings were largely consistent with a previously conducted study, where the activation of Smad signaling pathway has been confirmed to contribute to the anti-tumor effect of CSMD1 gene in melanoma cells [19]. Moreover, our results provided ample evidences indicating that the overexpression of CSMD1 gene, which functioned as a GC suppressor, could abolish the cancer-promoting activity of miR-642b-3p. Following the *in vitro* researches, *in vivo* experiments confirmed the anti-tumor power of down-regulated miR-642b-3p and up-regulated CSMD1 gene in the context of GC.

## Conclusion

This study suggested that miR-642b-3p targeted and down-regulated CSMD1 GC. Subsequently, inhibition of miR-642b-3p was found to impede the proliferative activities of GC cells *in vitro* as well as the tumor growth *in vivo* through up-regulation of CSMD1 expression, which was involved in the activation of Smad signaling pathway. Basically, the study offers novel insights of the pathogenetic mechanism of GC. More importantly, the findings of this study reasonably characterize that miR-642b-3p can act as an oncomiR driving the metastasis of GC, proposing it as a promising biomarker for the potential novel therapeutic schemes for GC in the future.

## Supplementary Material

Supplemental Material

## Data Availability

The data that supports the findings of this study are available in the manuscript and supplementary materials.

## Abbreviations

GC (gastric cancer); mRNAs (messenger RNAs); 3’-UTRs (3’-untranslated regions); CSMD1 (CUB and sushi multiple domains protein 1); qRT-PCR (quantitative real-time polymerase-chain reaction; TBST (Tris-buffered saline with 0.1% (v/v) Tween 20); DAB (3,3’-diaminobenzidine tetrahydrochloride); EDDM3A (epididymal protein 3A); BSA (bovine serum albumin); cDNA (complementary DNA); DMEM (Dulbecco’s modified Eagle’s medium); ECL (enhanced chemiluminescence); BCA (bicinchoninic acid); NC (nitrocellulose); HRP (horseradish peroxidase); MUT (mutant); NC (negative control); PBS (phosphate-buffered saline); α-SMA (alpha smooth muscle actin); FN (fibronectin); PMSF (phenylmethylsulphonyl fluoride); PVDF (polyvinylidene fluoride); SP (streptavidin-peroxidase); RIPA (radioimmunoprecipitation assay); SDS-PAGE (sodium dodecyl sulfate-polyacrylamide gel electrophoresis); WT (wild-type); microRNAs (miRNAs or miRs); ATCC (American Type Culture Collection); PEI (polyethylenimine); pCDH (protocadherin); PAX (paired box containing); MEM (minimum essential medium); GFP (green fluorescent protein); RPMI (Roswell Park Memorial Institute); NC (negative control); MEM (minimum essential medium); SDS-PAGE (sodium dodecyl sulfate-polyacrylamide gel electrophoresis); GEO (Gene Expression Omnibus); FBS (fetal bovine serum); IgG (immunoglobulin G); ELISA (enzyme-linked immunosorbent assay); SPF (specific pathogen-free); GAPDH (glyceraldehyde-3-phosphate dehydrogenase); MMP (matrix metalloproteinase); DAPI (4’,6-diamidino-2-phenylindole); ANOVA (analysis of variance)
