# Supplementary material for: MicroRNA-642b-3p functions as an oncomiR in gastric cancer by down-regulating the CUB and sushi multiple domains protein 1/smad axis
Source: Bioengineered. 2022 Apr 12;13(4):9614–28. doi: 10.1080/21655979.2022.2056813 (PMC9208452; doi:10.1080/21655979.2022.2056813)
Supplement: Supplemental Material [file KBIE_A_2056813_SM9022.zip › supplementary/Supplementary Table 1.docx]

**Supplementary Table 1.** Primer sequences for qRT-PCR

| Gene | Forward primer (5'-3') | Reverse primer (5'-3') |
| --- | --- | --- |
| miR-642b-3p | GACACAUUUGGAGAGGGACCC | Universal reverse primer of the kit |
| Smad7 | TTCCTCCGCTGAAACAGGG | CCTCCCAGTATGCCACCAC |
| Smad4 | CTCATGTGATCTATGCCCGTC | AGGTGATACAACTCGTTCGTAGT |
| CSMD1 | TGGAGGAGATTCCAGTCGCT | GCATAGTTCGGATACCCGTGA |
| MMP-2 | TACAGGATCATTGGCTACACACC | GGTCACATCGCTCCAGACT |
| MMP-9 | TGTACCGCTATGGTTACACTCG | GGCAGGGACAGTTGCTTCT |
| U6 | GCGGCGGATGGACTATCATA | Universal reverse primer of the kit |
| GAPDH | GGAGCGAGATCCCTCCAAAAT | GGCTGTTGTCATACTTCTCATGG |

Note: miR-642b-3p, microRNA-642b-3p; Smad7, SMAD family member 7; Smad4, SMAD family member 4; CSMD1, CUB and SUSHI multiple domain protein 1; MMP-9, matrix metalloproteinase-2; GAPDH, glyceraldehyde-3-phosphate dehydrogenase.
